# Unmet need for family planning among rural married women in Ethiopia: What is the role of the health extension program in reducing unmet need?

**DOI:** 10.1186/s12978-022-01324-x

**Published:** 2022-01-21

**Authors:** Daniel Tadesse, Girmay Medhin, Getnet M. Kassie, Tegene Legese Dadi, Setegn Tigabu, Mekdes Demissie, Mussie Alemayehu, Mulusew J. Gerbaba, Bisrat F. Denberu, Alula M. Teklu

**Affiliations:** 1 MERQ Consultant PLC, Addis Ababa, Ethiopia; 2 Aklilu Lemma Institute of Pathobiology, Addis Ababa University, Addis Ababa, Ethiopia; 3 International Institute for Primary Health Care-Ethiopia (IIfPHC-E), Addis Ababa, Ethiopia; 4 School of Public Health, Hawassa University, Hawassa, Ethiopia; 5 WHO Collaborating Centre for Mental Health Research and Capacity Building, Department of Psychiatry, School of Medicine, College of Health Sciences, Addis Ababa University, Addis Ababa, Ethiopia; 6 School of Nursing and Midwifery, College of Health Sciences and Medicine, Haramaya University, Haramaya, Ethiopia; 7 School of Public Health, Mekele University, Mekele, Ethiopia; 8 Department of Epidemiology, Faculty of Public Health, Institute of Health, Jimma University, Jimma, Ethiopia; 9 Federal Ministry of Health, Addis Ababa, Ethiopia

**Keywords:** Unmet need, Family planning, Health extension program, Rural Ethiopia, Women

## Abstract

**Background:**

Ethiopia is striving to reduce unmet need for family planning (FP) and implementation of the health extension program (HEP) is one of the major actions that the country took to address health issues of rural communities including FP. However, there is limited published evidence demonstrating the role of HEP in reducing the unmet need of married rural women for FP. The aim of this study is to estimate the role of HEP in reducing unmet need for FP in rural Ethiopia.

**Methods:**

This paper is based on data extracted from a national rural HEP assessment that covered all regions of Ethiopia. We identified 4991 eligible married women both from agrarian and pastoralist settings. The role of HEP was measured by the exposure of eligible women to FP services through the implementation of HEP packages. We used descriptive statistics to summarize different variables and used logistic regression to model the unmet need for FP.

**Results:**

The overall prevalence of unmet need for FP among married rural Ethiopian women was 22.41%, contraceptive prevalence rate (CPR) was 44.60%, and the total demand for FP was 60.86%. Women exposed to HEP had a lower level of unmet need (4.82%), a higher demand for FP (37.78%) and a higher CPR (24.93%) compared to women unexposed to HEP. Having exposure to FP services (adjusted odds ratio (AOR) = 0.46, 95% confidence interval (CI) 0.37–0.59), having level IV Health Extension Workers (HEWs) in the catchment health post (AOR = 0.80, 95% CI 0.67–0.95) and older age are significantly associated with lower levels of unmet need for FP. Having more children (AOR = 2.11, 95% CI 1.67–2.65) and better awareness of the husband about the availability of FP services (AOR = 1.22, 95% CI 1.01–1.48) were associated with a higher likelihood of an unmet need for FP.

**Conclusion:**

The unmet need for family planning is high in rural Ethiopia in general and among women who do not have exposure to HEP packages in particular. Assigning a better-qualified health worker at the health post, reaching out to pastoralist women, maximizing opportunities to counsel rural women about FP during any contact with HEWs, and increasing positive attitudes of husbands towards FP use are likely to have positive impacts in reducing the unmet need for FP of rural women.

## Plain language summary

Family planning is a method that couples can use to limit the number of child or space the gap. Unmet need for family planning is defined as the percentage of reproductive age women who wants to space or limit the number of children but not currently using any family planning method. There is a huge proportion of eligible women have an unmet need for family planning in Ethiopia. The health extension program is one of the strategies to reach rural women to improve the health of the community. Although, family planning service is one of the packages in a health extension program and this study aimed to estimate the role of health extension program in reducing unmet need for family planning. About 4991 married women were asked about the family planning use, need and the place where they get the services. During the assessment the role of health extension program was assessed by different question. Some of the major assessment areas were women exposer to service, service availability, awareness and mode of service delivery. One fourth of the women have unmet need for family planning. The family planning utilization is still low. The contribution of the health extension program in family planning service is significant. Women exposed to HEP through level 4 health extension worker and older age are significantly associated with low level of unmet need FP. The unmet need for family planning is high in rural Ethiopia. This will inform the improvement and sustainability of the program.

## Background

United Nations Sustainable Development Goals (SDGs) have targeted universal access to reproductive health services including provision of family planning (FP) methods [1]. In line with the global initiatives, Ethiopia is committed to promoting use of modern FP methods and has designed various strategies and policies to do so [2]. Ethiopia’s Federal Ministry of Health (FMOH) has set targets to reduce the unmet need for FP from 22 to 17% and to increase the contraceptive prevalence rate (CPR) from 41 to 55% by the end of 2029. Some of the efforts designed to encourage FP use [3] include provision of FP counseling by front line health workers including Health Extension Workers (HEWs).

Since 2003, the Ethiopian government has been engaged in promotion of access to basic health services to increase geographical accessibility of health services through the Health Extension Program (HEP) [4]. The frontline HEWs are responsible for providing basic health services including counseling of reproductive age women on fertility and use of modern contraceptive methods. With the ambitious 10-year Health Sector Transformation Plan-II that ends in 2029, and the urban–rural disparity that exists, the HEP is expected to play a key role in improving CPR, increasing demand for FP and reducing unmet need for FP. Unmet need for FP is defined as women who are fecund and sexually active but are not using any method of contraception, and report not wanting any more children or wanting to delay the birth of their next child for at least 2 years [5, 6]. This health indicator provides information on the gap between women’s intention to use FP and actual use of FP [7]. It is believed that the unmet need for FP can be reduced through providing FP in the primary health care setting through HEWs.

The implementation of FP in the primary health care setting was intended to increase access to FP options and contraceptive use among rural women [8]. The evidence shows that there is an increase of CPR and a decrease in unmet need for FP over time [9]. However, the current CPR among all women in rural Ethiopia (38.2%) and unmet need for FP among married rural women (24.4%) is not in line with the targets of the Health Sector Transformation Plan-II [9, 10]. Studies conducted in different parts of Ethiopia indicated that HEWs have an important role in providing maternal and child health services [11–13]. Despite this evidence, there is no national level evidence that shows the extent to which services from HEWs result in increased FP use. Such evidence will have significant value in informing policy makers who must decide whether to expand or maintain existing strategies and whether to expand the lessons to other pressing public health issues.

Furthermore, there is still a high level of unmet need for FP (22%) in Ethiopia [9, 14–16] which is higher among rural women than among urban women [17, 18]. However, there is limited information to quantitatively substantiate the level of unmet need in rural settings where the majority of people of the country reside with many challenges. Although HEP coverage has expanded, studies in limited geographic areas of the country showed high demand for FP and low CPR, implying high unmet need for FP. The aim of this study is to estimate the role of HEP in reducing unmet need for FP among rural Ethiopian women.

## Methods

### Study settings and context

During data collection Ethiopia was administratively divided into nine regional states (i.e. Tigray, Afar, Amhara, Oromia, Somali, Benishangul-Gumuz, Gambela, Harari, and SNNPR) and two city administrations (i.e. Addis Ababa and Dire Dewa). Each region is divided into woreda and then each woreda is divided into kebele which are the lowest government administrative structures. The study covered all the nine regional states where the livelihood is agrarian or pastoralist. There are three tiers of health delivery systems in Ethiopia, namely, primary, secondary and tertiary levels. The primary level consists of primary healthcare units (health posts and health centers) and primary hospitals. Health posts are staffed primarily with two HEWs who are recruited based on nationally agreed criteria that include residence in the village, capacity to speak local language, graduation from 10th grade, and willingness to remain in the village and serve communities. The HEWs go through a year of training with both theoretical and practical components. The HEP, which is implemented at a grassroots level by HEWs, was originally designed to enhance the primary health services in rural areas through an innovative community-based approach that focuses on prevention, healthy living and basic curative care. Family planning is one of the 18 packages of HEP that is expected to be delivered by HEWs at the health post level [19].

### Data source and study participants

Data used in this paper was extracted from a national rural HEP assessment which was conducted by MERQ consultancy PLC from October 2018 to September 2019. The assessment collected field data from March to May 2019. The assessment used multistage stratified sampling to select 62 districts or woredas from strata created by combining region with livelihood categories, three kebeles nested within each study woreda, and 34 households in each study kebele. The data was collected electronically using Open Data Kit (ODK). To develop this paper we extracted the data from married, rural, and aged 15–49 years from the national rural HEP assessment data base [20].

### Measurements

#### Outcome variable

The primary outcome variable is an unmet need for family planning. It is defined as “the proportion of women who (1) are not pregnant and not postpartum amenorrhoeic and are considered fecund and want to postpone their next birth for 2 or more years or stop childbearing altogether but are not using a contraceptive method, or (2) have a mistimed or unwanted current pregnancy, or (3) are postpartum amenorrhoeic and their last birth in the last 2 years was mistimed or unwanted.” [6, 21].

#### Primary exposure variable

Our primary variable of interest is exposure of women to FP services through HEP. It is a composite variable defined using five items, namely, whether a woman: (1) was aware of the presence of FP services in the health post, (2) received a home visit by a HEW in the last 1 year, (3) received FP education at home by the HEWs, (4) visited a health post in the last 1 year, and (5) received any counselling about FP. The response for each question was either yes or no. If a woman responded “yes” to at least one of the above five items, it was considered as “having exposure to FP service through HEP” and if a woman responded “no” to all of the five items then she was categorized as “not having exposure to FP service through HEP”.

#### Other covariates

Covariates include sociodemographic variables such as a woman’s age, number of children in the household, wealth index of the household (categorized into five categories as “Lowest quantile, lower quantile, middle quantile, higher quantile, and highest quantile”), maternal educational status (categorized into “no formal education” and “attend formal education”), a woman’s knowledge about FP, and livelihood (categorized as agrarian for a main livelihood of growing crops/farming or pastoralist for a main livelihood of livestock farming). Other covariates (further described below) were availability of health workers, the health post’s readiness for FP services, the qualifications of health workers at the health post, and women’s awareness about FPs.

#### Health post readiness for FP service

This variable was computed from the responses given to the following variables (a) availability of contraceptives supply and equipment in the health post and, (b) availability of FP services in the health post. The allowable response for each of the composite variables was yes or no. A health post was categorized as a “ready health post” for FP services if it had a response of “yes” for the two items. A health post was categorized as “not ready” if it received a “no” response for either item.

#### Qualification of health workers assigned to work at health post

This variable was measured by reviewing the professional qualifications of HEWs at the health post at the time of data collection. The response was categorized in to three levels, namely, level III HEWs (basic skills to provide services at a health post), level IV HEWs (1 year additional course after graduation of level III) and clinical nurse or midwife. We used the label “level III HEWs” for health posts who have level III HEWs as the maximum qualification. If the health post has at least one level IV HEW we categorized it as “level IV”. Health posts that have either clinical nurse/nurse or midwife were labelled as “clinical nurse or midwife”.

#### Women’s knowledge of modern FP

Knowledge of women about FP was collected by asking the woman if she had heard about a total of nine modern FP methods (male condom, female condom, implants, IUCD, female sterilization, male sterilization, injectable, pills and Lactational Amenorrhea Method (LAM)). The response categories for each question were either 0(no) or 1(yes). Finally, the responses on the nine questions were summed to create one continuous variable that measures knowledge of married women on modern FP.

#### Number of children

This variable document the number of living children the woman had during the data collection time. During data analysis it was coded in to three categories: ‘1–2’, ‘3–4’ and ‘5 or more’.

### Data quality assurance

Intensive training that lasted for 10 days was given for data collectors and their supervisors. In addition, regular supervision at the field during data collection was done to ensure data completeness and consistency of data. All possible data checking rules and logical checks were also implemented in the electronic data capturing template.

### Data analysis

Electronically collected data were exported to STATA version 15 for analysis. The complex nature of sampling and unequal probability of recruiting study participants were addressed by using weighting in every analysis. Descriptive statistics (frequencies and percentages) were used to summarize categorical variables, and mean with standard deviation was used to summarize continuous variables. Logistic regression was used to model the unmet need for FP and to investigate the effect of HEP. Unadjusted and adjusted odds ratios (OR) and a 95% confidence interval (CI) around the estimates were reported as measures of effect.

## Results

Out of 4991 eligible study participants, the majority were in the age range of 21–34 years, the mean age was 31 years (SD = 8), and the majority had no formal education (63.69%) (Table 1).

**Table 1:** Sociodemographic characteristics of study participants in Ethiopia, 2020

Background characteristics	Women	
	Weighted percent	Unweighted number
Age in years		
15–20	17.96	1044
21–34	41.97	2152
35–49	40.07	1795
Education status		
No formal education	63.69	3457
Attend formal education	36.31	1534
Wealth		
Lowest quantile	14.95	933
Lower quantile	19.49	999
Middle quantile	21.43	996
Higher quantile	23.46	1037
Highest quantile	20.67	1026
Current number of children		
1–2	28.29	1183
3–4	32.42	1419
5 and above	39.29	1650
Region		
Tigray	4.23	583
Afar	0.33	333
Amhara	26.68	1129
Oromia	47.74	1478
Somali	2.14	895
Benishangul-Gumuz	0.29	455
SNNPR	18.39	1075
Gambela	0.09	326
Harari	0.09	450
Livelihood		
Agrarian	95.37	3346
Pastoralist	4.63	1645

### Family planning service utilization, unmet need and demand

**Fig. 1:**
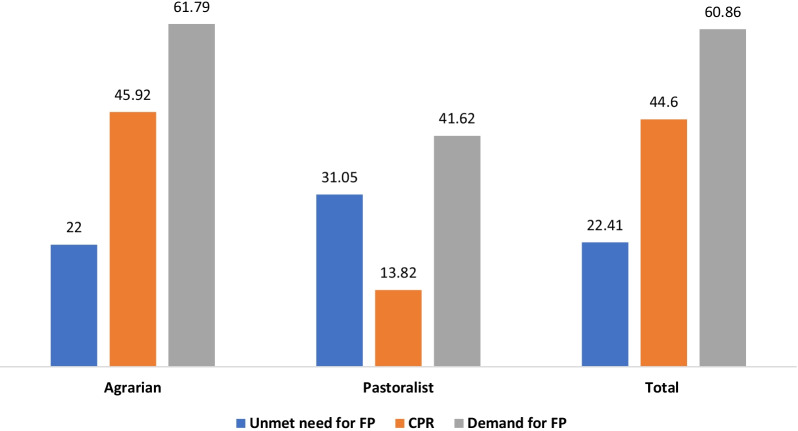
Unmet needs for family planning (FP), contraceptive prevalence rate (CPR) and demand for FP of married rural women aged 15–49 years by livelihood in Ethiopia, 2020

**Fig. 2:**
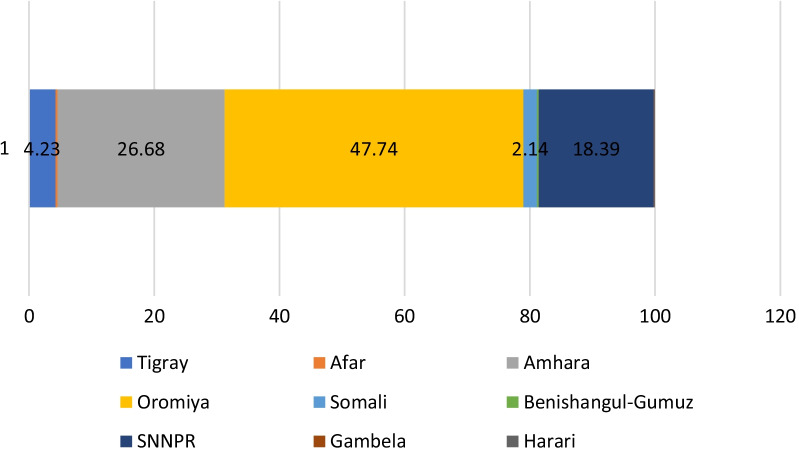
Unmet need for family planning, married women aged 15–49 by region in Ethiopia, 2020

The overall unmet need for FP among married women was 22.41%, the contraceptive prevalence rate (CPR) was 44.60% and total demand for FP was 60.86%. Women living in pastoralist settings had higher unmet needs (31.1%) and lower demand for FP (41.6%) compared to those in agrarian settings (Fig. 1). Unmet need is higher in Somali and Gambela compared to the other regions. Demand for FP was higher in the Amhara, Oromia and Beshagul-Gumuz regions (Fig. 2).

### Exposure to HEP and health post readiness for family planning services

A very large proportion of study participants (92.5%) had exposure to FP services through HEP/HEWs, 64.0% of catchment health posts had level IV HEWs and 92.0% of the health posts were ready to provide FP services (Table 2).

**Table 2:** Health post readiness for family planning (FP) services and exposure of women to health extension plan (HEP) FP services in Ethiopia, 2020

Exposure variables	Unweighted number	Weighted percentage
Women exposure to FP service through HEP		
Yes	4238	92.55
No	753	7.45
Maximum qualification of health workers at catchment health post		
Level III	1637	28.3
Level IV	2293	63.9
Clinical nurse or midwife	1061	7.8
Health post readiness to provide FP service		
Yes	4263	92.1
No	728	7.9

**Fig. 3:**
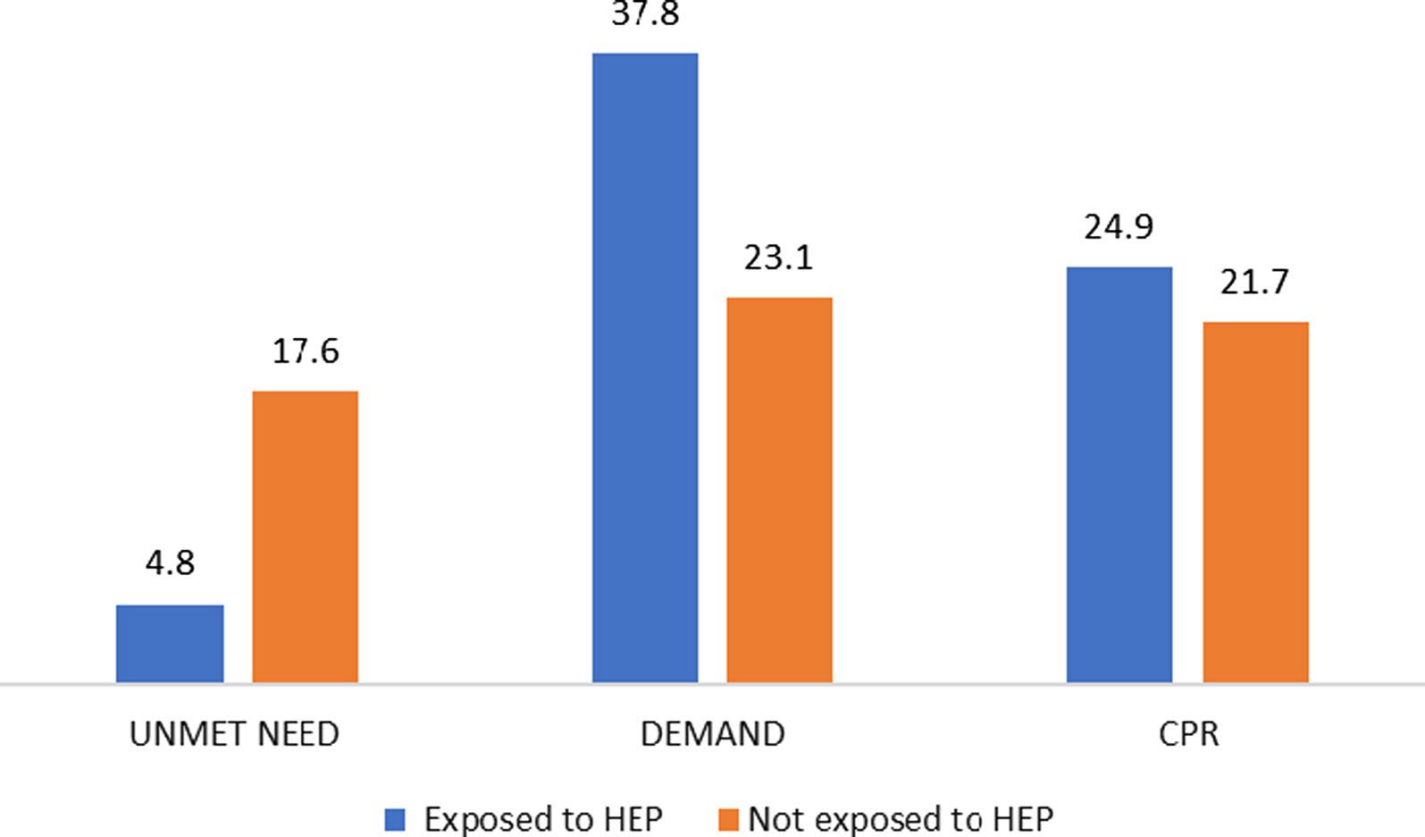
Percentage of married rural women aged 15–49 years exposed to Health Extension Plan services (HEP) with unmet needs for family planning (FP), demand for FP, and contraceptive prevalence rate, Ethiopia, 2020. *WDA* Woreda Development Army, *SMC* Social Mobilization Committee, *TV* Television, *HEW* Health Extension Worker

Women who had been exposed to HEP had lower levels of unmet need (4.82%), higher demand for FP (37.78%) and higher CPR (24.9%) compared to women with no exposure to HEP (Fig. 3).

### Knowledge of FP, current contraceptive use and reason for non-use of FP

**Fig. 4:**
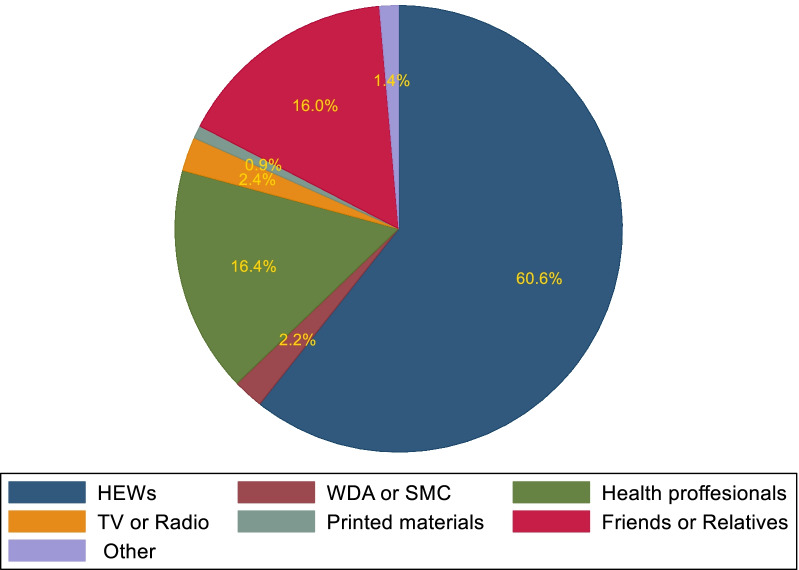
Primary sources of family planning information for married rural women in Ethiopia, 2020. *FS* Female sterilization

Among the study participant women, 87% knew at least one modern FP method. The best-known modern contraceptive methods were injectables (84.4%) followed by pills (75.5%). The least known FP methods were male sterilization (10.5%) and emergency contraceptives (14.1%). HEWs are the primary source of information about FP for married women (60.6%) (Fig. 4).

**Fig. 5:**
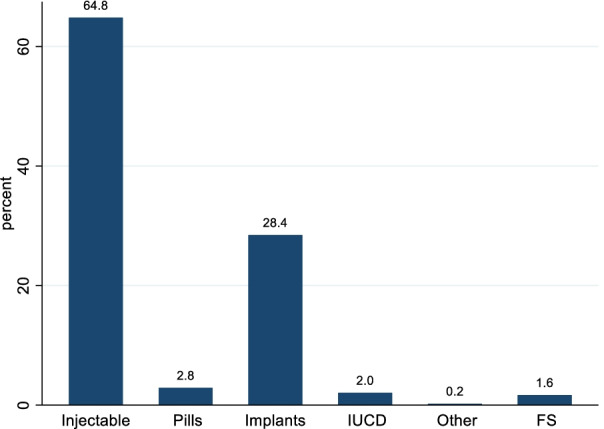
Methods of modern contraceptive used by married rural women in Ethiopia, 2020

The most commonly used modern contraceptive method was injectables (64.8%%) followed by implants (28.4%) (Fig. 5). The common reasons for not using contraceptives were being currently breast feeding (20.65%), not being allowed to use contraceptives by the religion of the respondent (16.4%) and fear of side effects (8.9%).

### Association of characteristics of women and unmet need for family planning

In the fully adjusted model women who had an exposure to HEP had 54% reduced odds of having an unmet need for FP compared to women who were not exposed to HEP (AOR = 0.46, 95% CI 0.37–0.59). Women living in catchment health post with level IV HEWs had 20% reduced odds of having an unmet need for FP compared to women who accessed health posts staffed with HEWs with a highest qualification of level III (AOR = 0.80, 95% CI 0.67–0.95), respectively. As the age of the women increases by 1 year the odds of unmet need decrease by 2% (AOR = 0.98, 95% CI 0.97–0.99). The odds of having an unmet need for FP for women whose household wealth status was in the highest quantile was 0.5% less compared to women whose household wealth index was in the lowest quantile. Women who had the highest numbers of living children (> 5) had 2 times increased odds of having an unmet need for FP compared to women who had 1–2 children (AOR = 2.11, 95% CI 1.67–2.65). Younger age, lower wealth, more children and husband awareness about FP services were significantly associated with unmet need for FP (Table 3).

**Table 3:** Association of characteristics of study participants with unmet need for family planning in rural Ethiopia, 2020

Variables	Weighted percentage with unmet need for FP	Unweight total number with unmet need for FP	COR (95% CI)	AOR (95% CI)
Women exposed to FP services through HEP	21.52	3295	0.56 (0.47, 0.66)	0.46 (0.37, 0.59)*
Maximum qualification of health workers				
Level III HEWs	Ref	Ref	Ref	Ref
Level IV HEWs	21.57	1785	0.73 (0.64, 0.85)	0.80 (0.67, 0.95)*
Clinical nurse or midwife	26.08	73.92	0.72 (0.35, 0.89)	0.90 (0.73, 1.13)
Health post ready to provide FP service	22.72	3269	0.60 (0.49, 0.73)	1.02 (0.75, 1.39)
Age in year				0.98 (0.97, 0.99)*
Women have formal education	17.34	82.66	0.72 (0.62, 0.83)	1.10 (0.92, 1.32)
Wealth				
Lowest quantile	Ref	Ref	Ref	Ref
Lower quantile	26.46	749	0.69 (0.56, 0.84)	0.86 (0.68, 1.08)
Middle quantile	22.81	761	0.64 (0.52, 0.78)	0.76 (0.60, 0.96)*
Higher quantile	20.62	812	0.57 (0.47, 0.70)	0.69 (0.54, 0.87)*
Highest quantile	18.19	841	0.46 (0.37, 0.56)	0.52 (0.41, 0.67)*
Women live in pastoralist area	31.05	1202	1.26 (1.10, 1.44)	1.05 (0.87, 1.26)
Women are knowledgeable about modern FP methods	21.84	3407	0.74 (0.61, 0.90)	0.76 (0.58, 1.01)
Current number of children				
1–2	Ref	Ref	Ref	Ref
3–4	22.95	1077	1.28 (1.06, 1.55)	1.29 (1.04, 1.59)*
5 and above	29.15	1138	1.82 (1.52, 2.17)	2.11 (1.67, 2.65)*
Husbands are aware of the availability of FP services at health post	21.73	3001	0.95 (0.82, 1.12)	1.22 (1.01, 1.48)*

*COR* crude odds ratio, *AOR* adjusted odds ratio, *FP* family planning, *HEP* Health Extension Plan, *HEW* Health Extension Workers

^*^Statistically significant

## Discussion

Among rural married women in Ethiopia the unmet need for FP is high and the CPR is low compared to national goal. Most of the women have exposure to HEP/HEWs and most of the health posts were ready to provide FP services. Women who are exposed to HEP have lower levels of unmet need, and higher contraceptive utilization, as compared to women with no HEP exposure. Moreover, the presence of level IV HEWs at the health post significantly contributed to the reduction of local women’s unmet need for FP compared to women who lived in a catchment health post with no level IV HEWs.

As per the national strategic plan Ethiopia is supposed to increase CPR to 55% and to reduce the unmet need for FP to 17% by the end of 2029 [10]. However, the country is still behind the target even though it has progressed and strived to increase contraceptive utilization and to decrease unmet needs. In the last two decades contraceptive utilization increased from a very low base of 8% in 2000 to 44.6% as reported in this study [9, 22]. The current finding is in line with studies conducted in different parts of Ethiopia which range from CPR of 19.1% to 31.4% [17, 23]. In the 2016 Ethiopia Demographic and Health Survey unmet need for FP in rural Ethiopia was 25%, which is not significantly different from the current finding although the two studies have a 4 year difference [9]. The high unmet need for FP and low contraceptive utilization might be attributed to deep rooted cultural influences due to the dominance of husbands and to religiosity. The dominance of husbands combined with the very low literacy level of women results in lack of open discussion about FP within married couples. Moreover, deep rooted cultural influences in which the culture encourage the dominance of husbands, deter women from controlling their own reproductive health [24]. The hurdles in the health system make their own contributions to high unmet need and low FP services utilization in Ethiopia [25]. Poor readiness of health facilities and poor quality of FP services are the major supply side barriers to reducing unmet need for FP, although the majority of health posts in the current study were ready for FP services. Despite the presence of good progress in reducing unmet need and increasing FP use in Ethiopia, this progress is not yet accompanied by an increase in the quality of FP counseling [26, 27]. Efforts focused on rural women may have a significant impact in reducing unmet need for FP and reaching Ethiopia’s 2029 target.

The implementation of HEP was envisioned to have an important role in the improvement of maternal and child health services. Increasing CPR and decreasing unmet need for FP are major activities of the HEWs [13]. The current findings showed that exposure to HEP is significantly associated with reduced unmet need for FP. This is linked to the fact that HEWs create awareness and contribute to changing women’s’ attitudes about FP. Increased awareness and attitude change of women create a demand for FP, and make rural women understand the different types of FP options. In line with this finding, previous studies showed that HEWs have a positive role in improving FP services in different parts of Ethiopia [12, 13, 28]. The HEWs improve FP service provision through delivering of appropriate information about the different available services to the mothers at the community and household level [13]. These findings imply two things: (1) increasing the knowledge of women about FP is the best way to decrease unmet need and to increase FP use; (2) strengthening the outreach and home visits of the HEP contribute to decreasing unmet need for FP. Thus, good implementation of HEP has a significant role in decreasing the unmet need for FP among rural women.

Another important finding in the current study is that the presence of level IV HEWs at the health post contributed significantly to reducing the unmet need for FP. While the availability of HEWs in the health post and their provision of FP services is crucial to reduce unmet need and to increase FP use, having a better qualified health care providers like level IV HEWs has a positive impact on the provision of services. One study conducted elsewhere in Ethiopia showed that level IV HEWs have better knowledge and skill in performing long acting FP methods than level III HEWs, which allows the level IV HEWs to make available more options of FP methods for their clients [29]. Thus, strengthening the capacity of HEWs by upgrading their qualifications has the potential for creating favorable ground for married women to get more options of FP methods [30], to decrease unmet need, and to increase FP use.

## Conclusion

The current study shows that unmet need for FP among women in rural Ethiopia is high and contraceptive service use is low as compared to the 2029 national target. Exposure of women to HEP through different platforms, having level IV HEWs at health posts, women being relatively older, women having a smaller number of children, and women having a higher household wealth index are associated with a reduced level of unmet need for FP. Coordinated activity is needed to increase the total demand for FP. Upgrading the qualifications of HEWs at health posts would be vital to reducing the unmet need for FP. Addressing basic socio-demographic variables (such as promoting educational attainment and increased household wealth) among married women would also help to reduce the unmet need for FP and increase total demand for FP.

### Strength and limitation

One of the strengths of the paper our use of nationally representative survey to extract data from which the findings reported in this paper are generated. However, there are number of limitations that any reader should have in mind while interpreting the findings. This paper has used data collected using a cross-sectional study design and temporal relations could not be established for the observed associations. Some of the study findings might have been affected by recall bias because the data was dependent on the retrospective recall of mothers. More over possibility of social desirability bias cannot be ignored. We might have missed to assess other influential factors that have a potential to reduce unmet need for family planning.

## Data Availability

Data will be available upon request to the corresponding author.

## Abbreviations

AOR: Adjusted odds ratio
CI: Confidence interval
CPR: Contraceptive prevalence rate
EPHI: Ethiopian Public Health Institute
FMOH: Federal Minster of Health
FP: Family planning
HEP: Health extension program
HEW: Health Extension Workers
IRB: Institutional Review Board
MERQ: Monitoring Evaluation Research and Quality improvement
OR: Odds ratio
SDG: Sustainable Development Goals
SNNPR: South Nation Nationality and People’s Region
